# Expression of aromatase and estrogen receptor alpha in chondrosarcoma, but no beneficial effect of inhibiting estrogen signaling both *in vitro *and *in vivo*

**DOI:** 10.1186/2045-3329-1-5

**Published:** 2011-07-25

**Authors:** Danielle Meijer, Hans Gelderblom, Marcel Karperien, Anne-Marie Cleton-Jansen, Pancras CW Hogendoorn, Judith VMG Bovée

**Affiliations:** 1 Department of Pathology, Leiden University Medical Center, Leiden, The Netherlands; 2 Department of Clinical Oncology, Leiden University Medical Center, Leiden, The Netherlands; 3 Department of Tissue Regeneration, MIRA, Institute for Biomedical Technology and Technical Medicine, University of Twente, Enschede, The Netherlands

## Abstract

**Background:**

Chondrosarcomas are malignant cartilage-forming tumors which are highly resistant to conventional chemotherapy and radiotherapy. Estrogen signaling is known to play an important role in proliferation and differentiation of chondrocytes and in growth plate regulation at puberty. Our experiments focus on unraveling the role of estrogen signaling in the regulation of neoplastic cartilage growth and on interference with estrogen signaling in chondrosarcomas *in vitro *and *in vivo*.

**Methods:**

We investigated the protein expression of estrogen receptor alpha (ESR1), androgen receptor (AR), and aromatase in tumor specimens of various chondrosarcoma subtypes, and (primary) chondrosarcoma cultures. Dose-response curves were generated of conventional central chondrosarcoma cell lines cultured in the presence of 17β-estradiol, dihydrotestosterone, 4-androstene-3,17 dione, 4-hydroxytamoxifen, fulvestrant and aromatase inhibitors. In a pilot series, the effect of anastrozole (n = 4) or exemestane (n = 2) treatment in 6 chondrosarcoma patients with progressive disease was explored.

**Results:**

We showed protein expression of ESR1 and aromatase in a large majority of all subtypes. Only a minority of the tumors showed few AR positive cells. The dose-response assays showed no effect of any of the compounds on proliferation of conventional chondrosarcoma *in vitro*. The median progression-free survival of the patients treated with aromatase inhibitors did not significantly deviate from untreated patients.

**Conclusions:**

The presence of ESR1 and aromatase in chondrosarcoma tumors and primary cultures supports a possible role of estrogen signaling in chondrosarcoma proliferation. However, our *in vitro *and pilot *in vivo *studies have shown no effect of estrogen-signaling inhibition on tumor growth.

## Background

Chondrosarcomas of bone are malignant cartilage-forming tumors which are highly resistant to conventional chemotherapy and radiotherapy [1,2]. However, recently various promising targets were discovered and the exploration of suitable therapies continues [3,4]. Conventional chondrosarcomas represent about 90% of all chondrosarcomas. Most conventional chondrosarcomas are located in the medullar cavity of the bone and are called central chondrosarcoma. About 15% of conventional chondrosarcomas arise from the surface of bone and are designated as peripheral chondrosarcomas. Conventional chondrosarcomas often show local destructive growth and the high-grade tumors commonly metastasize [5].

Besides conventional chondrosarcoma, several rare chondrosarcoma subtypes are defined, together constituting 10-15% of all chondrosarcomas. Dedifferentiated chondrosarcoma (10%) is a tumor containing a high-grade dedifferentiated non-cartilaginous sarcoma next to a usually low-grade malignant well-differentiated cartilage-forming tumor, with a sharply defined junction between the two components. It bears a poor prognosis and no targets for therapy have been reported so far [6]. Mesenchymal chondrosarcoma (2%) is a highly malignant lesion occurring in the bone and soft tissue of relatively young patients. The tumor consists of differentiated cartilage mixed with undifferentiated small round cells and usually follows an aggressive course with a high rate of distant metastases, and a 5-year overall survival of 55% [7]. Clear cell chondrosarcoma (2%) is a low-grade malignant tumor, which rarely metastasizes, but commonly recurs after curettage. About 15% of the patients die as a result of the disease [8]. The lack of efficacious treatment for all different subtypes of chondrosarcomas emphasizes the need to identify new treatment strategies.

One of the potential targets for therapy is the estrogen-signaling pathway. Mutations in *ESR1 *and *CYP19A1*, the gene for aromatase, demonstrated an important role for estrogen in the proliferation and differentiation of chondrocytes in the epiphyseal growth plate [9]. Estrogen induces the pubertal growth spurt, and at the end of puberty growth plate fusion [10]. Furthermore, osteochondromas, the benign precursors of peripheral chondrosarcomas, stop growing at the end of puberty, suggesting an inhibitory effect of estrogens on these tumors. In addition, ESR1 and ESR2 expression has been shown to be a common phenomenon in chondrosarcomas [11,12]. In a previous study, our group also demonstrated functional activity of the estrogen-producing enzyme aromatase in chondrosarcoma cells *in vitro *[11]. These results indicated that the ESR signaling pathway might be a potential target for endocrine treatment of metastatic or irresectable chondrosarcoma.

For already three decades endocrine therapy plays a crucial role in the treatment of women with hormone-responsive breast cancer. Breast cancer and chondrosarcomas were found to occur relatively frequently in the same patient. A population-based study by Odink et al. implicated a 7.62 times increased risk for the same female patient to have both breast cancer and a cartilaginous tumor [13]. The mean age of onset in patients with breast cancer as the first tumor and chondrosarcoma as a second tumor is nearly 10 years earlier than breast cancer in general [13]. These observations may suggest a genetic trait. Remarkably, the expression of ESR1 was significantly higher in breast cancer associated with chondrosarcoma [14].

The two strategies used for endocrine treatment are blockade of ESR1 using selective estrogen receptor modulators/downregulators like tamoxifen and fulvestrant, and deprivation of estrogen production by inhibiting aromatase with anastrozole, letrozole, and exemestane. In our above-mentioned study, we showed that the aromatase activity and proliferation of chondrosarcoma cells slightly decreased after addition of the aromatase inhibitor exemestane [11]. In our present study, we focused on further unraveling the role of estrogen in the regulation of neoplastic cartilage growth in a larger cohort of various chondrosarcoma subtypes, including conventional central and peripheral chondrosarcoma as well as dedifferentiated, mesenchymal, and clear cell chondrosarcoma. Moreover, using a larger set of drugs targeting the estrogen-signaling pathway we investigated whether interference with estrogen signaling could inhibit chondrosarcoma growth. We aimed to validate and expand our previous *in vitro *data by measuring the effects of estrogens, androgens, tamoxifen, fulvestrant, and aromatase inhibitors on the proliferation of various chondrosarcoma cell cultures. Furthermore, we explored the efficacy of aromatase inhibitors in a set of patients with metastatic or locally advanced chondrosarcoma.

## Methods

### Tumor tissue

All specimens in this study were handled according to the ethical guidelines described in "Code for Proper Secondary Use of Human Tissue in The Netherlands" of the Dutch Federation of Medical Scientific Societies. Conventional central and peripheral chondrosarcoma, and the rare subtypes dedifferentiated, mesenchymal, and clear cell chondrosarcoma were selected based on accepted clinicopathological and radiological criteria [15]. In total, formalin-fixed paraffin-embedded (FFPE) specimens from 175 patients, including the 6 patients in our pilot study, were collected from the archives of the Department of Pathology, LUMC, The Netherlands (n = 100), Nuffield Department of Orthopaedic Surgery, University of Oxford, UK (n = 7), Institute of Orthopaedics and Musculoskeletal Science, UCL, UK (n = 22), Laboratory of Oncologic Research, ROI, Italy (n = 30), Department of Pathology, RH, Denmark (n = 9), Department of Pathology, Medizinische Universität Graz, Austria (n = 7). All were primary tumors except for three clear cell chondrosarcomas and six mesenchymal chondrosarcomas, from which only recurrences were available. Clinicopathological data are shown in table 1. Histological grading was performed according to Evans [5].

**Table 1:** Clinicopathological data of the 175 formalin-fixed paraffin-embedded cartilaginous tumors

	EC	CS	OC	PCS	DDCS	CCS	MCS
**Total number of tumors**	**3**	**46**	**10**	**28**	**42**	**23**	**23**
Grade I	-	16	-	12	-	-	-
Grade II	-	18	-	13	-	-	-
Grade III	-	12	-	3	-	-	-
							
Male	1	22	6	17	21	17	8
Female	2	23	4	10	21	6	15
Enchondromatosis/MO	0	1	5	10	-	-	-
Median age yrs (range)	38 (37-50)	51 (20-79)	14.5 (6-24)	38 (16-61)	66 (26-85)	43 (20-79)	29.5 (15-70)

Abbreviations: enchondroma (EC), conventional central and peripheral chondrosarcoma (CS and PCS), osteochondroma (OC), dedifferentiated chondrosarcoma (DDCS), clear cell chondrosarcoma (CCS), and mesenchymal chondrosarcoma (MCS).

### Tissue microarray (TMA) construction

Of the rare chondrosarcoma subtypes we constructed TMAs using a TMA Master (3DHISTECH Ltd, Budapest, Hungary). TMAs contained 2 mm cores of each sample, in triplicate. From the dedifferentiated chondrosarcomas we included both the well differentiated and the dedifferentiated components. The clinical details are outlined in Table 1. Normal non-decalcified liver, kidney, and tonsil samples were included on the TMAs for orientation purposes and as internal positive controls.

### Immunohistochemistry (IHC)

Details of the primary antibodies used for immunohistochemistry are described in Table 2. As negative controls, slides were incubated in PBS/BSA 1% without primary specific antibodies. AR and aromatase immunohistochemical stainings of the patient material were semi-quantitatively scored for nuclear and cytoplasmic staining respectively. Scores were given for intensity (0 = absent, 1 = weak, 2 = moderate, 3 = strong) and for the percentage of positive cells (0 = 0%, 1 = 1-24%, 2 = 25-49%, 3 = 50-74%, and 4 = 75%-100%). To avoid tumors with single positive cells being regarded as positive, a cut-off level of a total sum ≥4 was applied. ESR1 was scored for nuclear staining, with positivity defined as ≥10% (weakly) positive cells, according to standard clinical procedures for scoring ESR1-positive breast cancer [16,17]. Scoring was performed by two independent observers without knowledge of the clinicopathological data. For dedifferentiated chondrosarcoma, the well differentiated and the dedifferentiated component were scored separately. Likewise, for mesenchymal chondrosarcoma both the cartilaginous areas and the small cell component were evaluated.

**Table 2:** Procedures and details of the primary antibodies used for immunohistochemistry

Protein	Origin	Number	Dilution	Species	Antigen retrieval	Blocking	Positive control
ESR1	Invitrogen/zymed	18-0174Z	1:200	Rabbit	Tris-EDTA	30' 5% ELK milk	breast cancer
aromatase	Abcam	Ab18995	1:300	Rabbit	Citrate	30' 5% ELK milk	placenta
AR	Dako	AR441	1:200	Mouse	Tris-EDTA	-	cervix stroma

### Cell cultures and conditions

Chondrosarcoma cell lines SW1353 (ATCC, Manassas, VA), OUMS27 [18], CH2879 [19], and JJ012 [20], and breast cancer cell line ZR-75-1 [21] were cultured in RPMI 1640 supplemented with 10% heat-inactivated fetal bovine serum (FBS) (Gibco, Invitrogen Life-Technologies, Scotland, UK) (Table 3). ZR-75-1 cultures were additionally supplemented with 1 nmol/L 17β-estradiol (E_2_) (Sigma). Two chondrosarcoma primary cultures, L835 and L869, were generated as described previously [11] and were cultured in collagen I-coated culture flasks in RPMI 1640 supplemented with 20% heat-inactivated fetal calf serum (Invitrogen), 2% penicillin/streptomycin (MP Biomedicals), and 1% glutamax (Invitrogen) (Table 3). Cells were grown at 37°C in a humidified incubator with 95% air and 5% CO_2_. The primary chondrosarcoma cultures expressed mRNA of at least two of the cartilaginous markers collagen 2, collagen 10, aggecan or SOX9 [11]. In addition, karyotyping of L835 and L869 by COBRA-FISH showed an aberrant number of chromosomes, thereby confirming their tumorigenic origin.

**Table 3:** Chondrosarcoma cultures

	Sample	Type	Grade	Gender	Age	Passage
1	SW1353	Cell line	II	F	72	18
2	CH2879	Cell line	III	F	35	31
3	OUMS27	Cell line	III	M	Na	22
4	JJ012	Cell line	II	M	39	10
5	L835	Primary culture	III	M	55	15
6	L869	Primary culture	II	M	52	18

### Protein detection in chondrosarcoma cell cultures

Four T75 culture flasks of 4 chondrosarcoma cell lines (SW1353, CH2879, OUMS27, JJ012) and 2 primary chondrosarcoma cell cultures (L869 and L835), and a positive control (ZR-75-1) were trypsinized and washed twice with cold PBS. Cells were formalin-fixed over night and subsequently embedded in paraffin. Using IHC, we determined ESR1 protein expression, as described above.

### Proliferation assays

To monitor the effects of the estrogen signaling pathway on chondrosarcoma cell proliferation we performed various experiments. An overview of all different conditions tested is given in Table 4. For the WST-1 assays with steroids and inhibitors, SW1353, CH2879, OUMS27, JJ012, L869, and L835 cells were seeded into collagen I-coated 96 wells plates (BD Biosciences) at a density of 1500 cells per well for the SW1353 and JJ012 cell lines and 5000 cells per well for the other cultures. The cells were plated in phenol red-free RPMI 1640 medium (Invitrogen) supplemented with 10% heat-inactivated charcoal-stripped FBS (Invitrogen). After 24 hours, serial dilutions of the steroids 17β-estradiol (Sigma), 4-androstene-3,17-dione (Sigma) and dihydrotestosterone (Fluka Analytical) (100 pM-1 μM), anti-steroids 4-hydroxytamoxifen (Sigma) and fulvestrant (Sigma) (1 nM-10 μM), aromatase inhibitors anastrozole, letrozole and exemestane (1 nM-10 μM) or combinations were added. The compounds were solved in ethanol. As vehicle control, ethanol was added with concentrations never exceeding 0.1%. All concentrations were tested at least in quadruplicate in a total volume of 100 μl. After 3 days, 10 μl proliferation reagent WST-1 (Roche Diagnostics) were added to each well, and the cells were returned to the incubator for three hours. Absorbance was measured at 450 nm with a Victor^3 ^Multilabel Counter 1420-042 (Perkin Elmer, MA, USA). Values were corrected for background, averaged and normalized to the vehicle-control cultures. FBS dependence was tested likewise for SW1353, CH2879, and OUMS27. For cell counting experiments, 24000 cells were seeded in a 24 wells plate. The experimental set up was identical to the proliferation assays with a total volume of 1 ml. Cells were counted after 3 and 7 days of treatment according to experiments previously published by our group [3,11].

**Table 4:** Experimental conditions tested

Test	Experimental conditions
Steroids	E2 (Fig 2A), ASD, DHT (100 pM-1 μM)
Inhibitors of estrogen signaling	OHT, Fulvestrant, Anastrozole, Letrozole, Exemestane (1 nM-10 μM)
Steroids combined with inhibitors	E2 (1 nM) with OHT or Fulvestrant (1 nM-10 μM) (Fig. 2B)
FBS	1%, 5%, 10% FBS alone and combined with E2 or ASD (100 pM-1 μM)
Timepoints of measurements	3 days, 7 days
Methods of measurement	WST1 viability assay, cell counting

Abbreviations: 17β-estradiol (E2), 4-androstene-3,17-dione, (ASD), dihydrotestosteron (DHT), 4- hydroxytamoxifen (OHT), Fetal bovine serum (FBS).

### Patients

Five patients with grade II or III conventional chondrosarcoma and one patient with dedifferentiated chondrosarcoma were treated with the aromatase inhibitors anastrozole 1 mg once daily (4 patients, including the patient with dedifferentiated chondrosarcoma) and exemestane 25 mg once daily (2 patients). Median age was 44 years (range 33-68); 3 patients had metastatic disease and 3 had locally advanced tumors. Tumor measurements and response evaluations were performed according to RECIST [22]. From the patients with conventional chondrosarcoma, FFPE tumor specimens were stained for ESR1 and aromatase protein.

## Results

### Expression of ESR1, aromatase, and AR in FFPE chondrosarcoma tumor specimens

**Figure 1:**
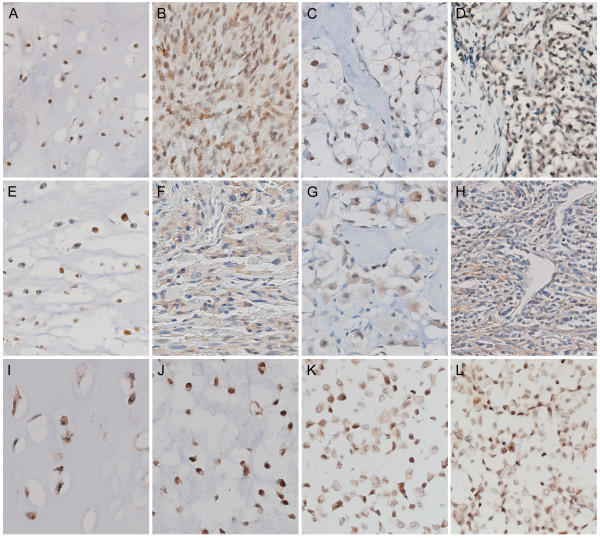
**Immunohistochemical staining of ESR1 and aromatase in various chondrosarcoma subtypes and cell lines**. Nuclear protein expression of ESR1 in well-differentiated (A) and dedifferentiated (B) components of dedifferentiated chondrosarcoma, clear cell chondrosarcoma (C), and mesenchymal chondrosarcoma (D). Cytoplasmic protein expression of aromatase in well-differentiated (E) and dedifferentiated (F) components of dedifferentiated chondrosarcoma, clear cell chondrosarcoma (G), and mesenchymal chondrosarcoma (H), and aromatase and ESR1 protein expression in conventional chondrosarcoma (I and J, respectively). ESR1 protein expression in the JJ012 and CH2879 chondrosarcoma cell lines (K and L). Magnification 200×.

Results of ESR1, aromatase, and AR immunohistochemical stainings on 175 FFPE tumor specimens are shown in Table 5. Expression for ESR1 and aromatase was detected in the majority of all subtypes (Table 5 and Figure 1). In conventional central and peripheral chondrosarcoma we observed immunoreactivity against ESR1 in 81% (34 out of 42) and 81% (21 out of 26) of the tumors, respectively. We observed ESR1 in 73% of the well differentiated and in 84% of the dedifferentiated component of dedifferentiated chondrosarcoma. Positive staining of the two components was strongly correlated. In mesenchymal chondrosarcomas, 67% of the small cell components were positive, versus 33% of the cartilaginous areas. Only a few strongly AR positive cells were detected in a minority of the chondrosarcomas of various subtypes. Aromatase protein, the enzyme responsible for the conversion of androstenedione and androgens to estrogens, was expressed in 86% (38 out of 44) and 93% (25 out of 27) of the central and peripheral chondrosarcomas respectively. Almost all well differentiated (97%) and dedifferentiated (89%) components of dedifferentiated chondrosarcoma were positive for aromatase. Of the cartilaginous area of mesenchymal chondrosarcoma 77% showed aromatase positivity versus 52% of the small cell component. In central chondrosarcoma no correlation with histological grade was observed with any of the proteins. In peripheral chondrosarcoma only 33% of the grade III tumors showed ESR1 expression. However, only three tumors were included in this group.

**Table 5:** Immunohistochemical staining of 175 FFPE samples of chondrosarcoma patients

	ESR1		aromatase		AR*	
**Enchondroma**	2/2	100%	3/3	100%	0/2	0%
						
**Central CS**	34/42	81%	38/44	86%	6/41	15%
grade I	11/15	73%	13/15	87%	1/13	8%
grade II	14/16	88%	15/17	88%	3/16	19%
grade III	9/11	82%	10/12	83%	2/12	17%
						
**Osteochondroma**	5/8	63%	6/8	75%	1/7	14%
						
**Peripheral CS**	21/26	81%	25/27	93%	4/28	14%
grade I	9/11	82%	11/12	92%	2/12	17%
grade II	12/13	92%	12/13	92%	1/13	8%
grade III	1/3	33%	3/3	100%	1/3	33%
						
**Dedifferentiated CS**						
well differentiated component	18/25	72%	31/32	97%	1/27	4%
dedifferentiated component	30/35	86%	34/38	89%	2/37	5%
						
**Clear cell CS**	15/22	69%	15/22	69%	0/22	0%
						
**Mesenchymal CS**						
small cell component	15/23	65%	12/23	52%	1/23	4%
cartilaginous areas	5/15	33%	10/13	77%	0/12	0%

* In positive samples only few strongly positive cells were observed.

### Estrogen responsiveness of central chondrosarcoma *in vitro*

**Figure 2:**
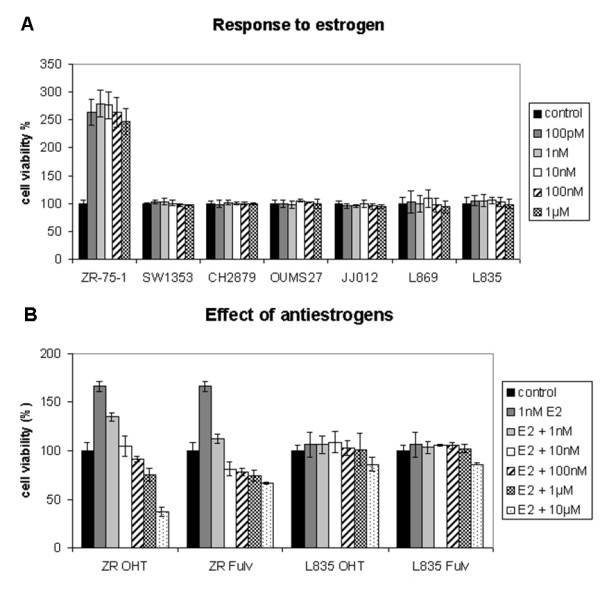
**Cell viability assays measuring the effect of estrogen and antiestrogens in chondrosarcoma cell lines**. A) Unlike breast cancer cell line ZR-75-1, chondrosarcoma cell lines (SW1353, CH2879, OUMS27, and JJ012) and primary cultures (L869 and L835) did not respond to E2 with increased proliferation; B) also none of these cultures responded to 4-hydroxytamoxifen (OHT), and fulvestrant (Fulv) in the presence of 1 nM E2. Only L835, which is representative, is shown.

SW1353, CH2879, OUMS27, JJ012, L869 and L835 were positive for ESR1 protein staining (Figure 1K, 1L and data not shown). Therefore, we investigated the effect of ESR-signaling modulation on the proliferation of chondrosarcoma cells *in vitro *by measuring the effect of steroids and clinical drugs inhibiting estrogen-signaling. We evaluated responsiveness of 4 central chondrosarcoma cell lines and 2 primary cultures to 3 different steroids (17β-estradiol, the estrogen precursor androstenedione, and the non-aromatizable androgen dihydroxytestosterone). The proliferation of the cells was not significantly influenced by any of these factors in the chondrosarcoma cell lines and primary cultures, whereas a clear response was observed in the proliferation rate of the ZR-75-1 breast-cancer cell line, which was used as a positive control (Figure 2Aand data not shown). We also tested three aromatase inhibitors (anastrozole, letrozole, and exemestane), and two estrogen-receptor antagonists/downregulators (4-hydroxytamoxifen and fulvestrant) and again no effect was shown in the chondrosarcoma cell lines and cultures under the different conditions described in Table 4 (Figure 2Band data not shown).

### Clinical results

In a pilot series, 6 consecutive patients with locally advanced or metastatic grade II or III conventional or dedifferentiated chondrosarcoma, for whom no standard treatment was available, were treated with aromatase inhibitors after informed consent. All tumors were radiologically progressive in the 6 months before initiation of therapy. All five conventional chondrosarcomas expressed ESR1 and aromatase protein, supporting the rationale of the treatment. The median progression-free survival was 5 months (range 4-10 months) in the conventional chondrosarcoma patients and 2 months in the patient with a metastatic dedifferentiated chondrosarcoma, which did not significantly deviate from untreated patients.

## Discussion

Chondrosarcoma of bone is a malignant cartilage-forming tumor of which distinct clinical and histological subtypes are recognized. So far, for locally advanced and metastatic chondrosarcoma no treatment options are available. Previous studies have demonstrated the presence of the ESR1 and activity of aromatase in conventional chondrosarcoma [11,12]. Furthermore, in 2005, our group showed an effect of estrogens and the aromatase inhibitor exemestane on the proliferation of chondrosarcoma cells *in vitro*, indicating that chondrosarcomas might be susceptible to hormonal therapy. In that study, *ESR1 *and *CYP19A1 *mRNA expression were demonstrated in a set of 23 conventional chondrosarcomas and 7 (primary) chondrosarcoma cultures. ESR1 protein expression was demonstrated in all 23 tumors tested. Addition of 17β-estradiol, 4-androstene-3,17-dione, and exemestane showed subtle effects on the proliferation of 2 cell cultures containing ESR1 and aromatase. After addition of 4-androstene-3,17-dione, an increase in proliferation was demonstrated. Proliferation was 131% of normal proliferation which decreased to 105% after inhibition with exemestane. A cell line lacking *ESR1 *and *CYP19A1 *did not show any response.

In the current study, we aimed to gain more insight into the possibility of treating chondrosarcoma patients with hormonal therapy by further investigating the expression of the hormone receptors ESR1 and AR, and of aromatase, the enzyme that mediates the last step in the biochemical formation of estrogen, in a larger set of conventional chondrosarcomas as well as three rare chondrosarcoma subtypes. In conventional chondrosarcoma, we furthermore monitored the effect of estrogen, the estrogen precursor androstenedione and the non-aromatizable androgen dihydrotestosteron, and various known estrogen signaling-inhibiting drugs on the progression of chondrosarcoma cells *in vitro*.

We demonstrated expression of ESR1 in a large proportion of various types of cartilaginous tumors. In conventional central and peripheral chondrosarcoma we observed immunoreactivity against ESR1 in 81% (34 out of 42) and 81% (21 out of 26) of the tumors, respectively. These results confirm and extend 2 previous studies in which the authors demonstrated nuclear expression of ESR1 in subsets of 23 [11] and 31 [12] conventional chondrosarcomas. Grifone et al. [12] suggested a decrease or loss in ESR1 expression in the higher grade or dedifferentiated chondrosarcomas. We observed such a trend in the peripheral chondrosarcomas, where only 33% of the high grade tumors show positive staining for ESR1. However, this group included only three tumor specimens. In central chondrosarcoma no correlation with grade was observed. Aromatase protein, the enzyme responsible for the conversion of androstenedione and androgens to estrogens, was expressed in 86% and 93% of the central and peripheral chondrosarcomas respectively, suggesting that tumors are capable of metabolizing estrogens from precursors. AR is another important target for hormonal therapy in for example prostate cancer. As androstenedione is a steroid precursor for estrogens as well as androgens we also investigated the possibility of AR involvement in chondrosarcoma proliferation. However, AR nuclear protein expression was observed only in a small number of cases with very few positive cells.

Besides conventional chondrosarcoma, several rare chondrosarcoma subtypes are defined. Despite aggressive therapy, approximately 90% of the patients with dedifferentiated chondrosarcoma die with distant metastasis, within 2 years after diagnosis of the disease [6,23]. The low-grade component and the highly malignant component display ESR1 protein expression in 72% and 86% of the samples respectively. Aromatase was observed in 97% and 89%, suggesting the presence of estrogens.

Mesenchymal chondrosarcomas are usually very aggressive with a strong tendency of local recurrence and distant metastases. Patients have a 5-year overall survival of 55% [7]. Although mesenchymal chondrosarcoma of bone is generally considered to lack sex predilection [24], Fanburg-Smith et al. [25] suggested a female predominance and raised the possibility of hormonal influence in the pathogenesis of this tumor. However, all their mesenchymal chondrosarcoma cases were ESR1 negative. In our study, in 65% (15 out of 23) of the mesenchymal chondrosarcomas the small cell component was positive for ESR1, while in 33% (5 out of 15) of the tumors also the cartilaginous areas were positive. Moreover, aromatase expression was observed in the small cells of 52% (12 out of 23) of the tumors, whereas the cartilage component demonstrated aromatase expression in 77% (10 out of 13). This might indicate that these tumors do have an active estrogen signaling pathway, which might be targetable by antiestrogens or aromatase inhibitors. Discrepant results may be explained by differences in ESR1 antibody and antigen retrieval protocols.

Clear cell chondrosarcoma is a low-grade variant of chondrosarcoma, which rarely metastasizes, but has a recurrence rate of 86% after curettage. About 15% of the patients die as a result of the disease [8]. We have observed ESR1 expression and aromatase expression each in 69% of the clear cell chondrosarcomas, suggesting that also these chondrosarcoma patients potentially might benefit from antiestrogen therapy and/or aromatase inhibition.

*In vitro *cell models to further study the effect of estrogen signaling on chondrosarcoma are available for conventional central chondrosarcoma only. No stimulation of proliferation of central chondrosarcoma cells was observed after addition of the non-aromatizable androgen dihydrotestosterone. This suggests no significant role for AR signaling in chondrosarcoma proliferation, which is consistent with the fact that very few tumors express AR.

In addition, in spite of positive immunohistochemical staining for ESR1 protein in all *in vitro *cell cultures, addition of 17β-estradiol, 4-androstene-3,17-dione or drugs targeting the estrogen-signaling pathway did not have a significant effect on the proliferation of the conventional central chondrosarcoma cell cultures. These results contradict our results published in 2005, where proliferation was stimulated by 17β-estradiol and 4-androstene-3,17-dione, and inhibited by exemestane [11]. Although we included an identical experimental set up, cell culture conditions are never 100% identical. For example, each batch of FBS contains different amounts of growth factors and other components which might influence experimental outcome. Also cell characteristics might have changed over time, resulting in passages insensitive to (anti)estrogens and aromatase inhibitors, as has been described before for certain breast cancer cell lines [26-28].

Breast cancer cell line ZR-75-1 is known to be completely dependent on estrogens for its proliferation, and proliferation can be fully inhibited by abrogating the estrogen-signaling pathway [29]. Although we previously demonstrated an effect of estrogen-signaling on chondrosarcoma cell proliferation, as compared to estrogen-dependent breast cancer cell line ZR-75-1 the effects in chondrosarcoma, if present, were very subtle. As a positive control, ZR-75-1 showed a 179% increase of proliferation upon addition of 1 nM 17β-estradiol, confirming a functional experimental setup, versus a previously demonstrated 55% increase in chondrosarcoma proliferation [11] and no significant increase in the current study. Both studies clearly indicate that, in contrast to estrogen-dependent breast cancer, chondrosarcoma proliferation is not fully dependent on estrogens.

Besides investigating estrogen dependence, we tested aromatase inhibitors which block estrogen production, and the effects of tamoxifen and fulvestrant which abrogate estrogen receptor function [30,31]. In the estrogen-dependent ZR-75-1 breast-cancer cell line proliferation was completely inhibited upon addition of tamoxifen and fulvestrant (Figure 2B). However, in the chondrosarcoma cell cultures, estrogen-signaling inhibition caused no effects on cell proliferation, suggesting that the mechanism driving proliferation in chondrosarcoma is different from the mechanism active in estrogen-dependent breast cancer. In chondrosarcoma, effects of estrogen are much more subtle and likely depend on the tissue culture conditions used, resulting in either marginal effects (in our previous study) or no effects at all.

In addition, the median time to progression in the clinical series was five months both before and after treatment. Therefore, we can conclude that aromatase inhibition was not effective in five conventional chondrosarcoma patients, nor in a patient with dedifferentiated chondrosarcoma. Although a formal prospective phase II trial would have been more suitable to prove (in)efficacy of this concept, we were not able to gain industry support without stronger preclinical data.

Since our study is limited to the effects of estrogen signaling on conventional central chondrosarcoma only, no conclusions can be drawn about the effects of estrogen signaling in the other chondrosarcoma subtypes. However, although we demonstrated the presence of aromatase and ESR1 in a majority of various chondrosarcoma subtypes, our *in vitro *data on conventional chondrosarcoma and our patient trial including one dedifferentiated chondrosarcoma patient suggest that effects of estrogen-signaling inhibition in other chondrosarcoma subtypes, if present at all, will be very small and that estrogen-signaling inhibition is unlikely to play a major role in chondrosarcoma management.

## Conclusions

In summary, we demonstrated the presence of the components involved in estrogen signaling in a large majority of chondrosarcomas. However, we could not demonstrate a significant effect of estrogen or inhibitors of estrogen signaling on cell proliferation and viability *in vitro *using central chondrosarcoma cell lines and primary cultures. Despite the previously presented and currently confirmed biological rationale, our *in vitro *and pilot clinical data suggest that an active estrogen-signaling pathway might just not play a pivotal role in the development and progression of conventional chondrosarcoma and do not support the further development of therapeutic strategies including inhibition of estrogen signaling in chondrosarcoma.

## Competing interests

The authors declare that they have no competing interests.

## Authors' contributions

DM carried out the experiments and drafted the manuscript. HG carried out the pilot patient study. MK, AMCJ and PCWH participated in the design of the study, the interpretation of data, and revision of the manuscript. JVMGB conceived of the study, and participated in its design and coordination, and helped to draft the manuscript. All authors read and approved the final manuscript.
